# Supplementary material for: A Mixture of Cervus elaphus sibiricus and Glycine max (L.) Merrill Inhibits Ovariectomy-Induced Bone Loss Via Regulation of Osteogenic Molecules in a Mouse Model
Source: Int J Mol Sci. 2023 Mar 2;24(5):4876. doi: 10.3390/ijms24054876 (PMC10003697; doi:10.3390/ijms24054876)
Supplement: Supplementary file 1 [file ijms-24-04876-s001.zip › ijms-2202575-supplementary.pdf]

Supplementary figure. 1

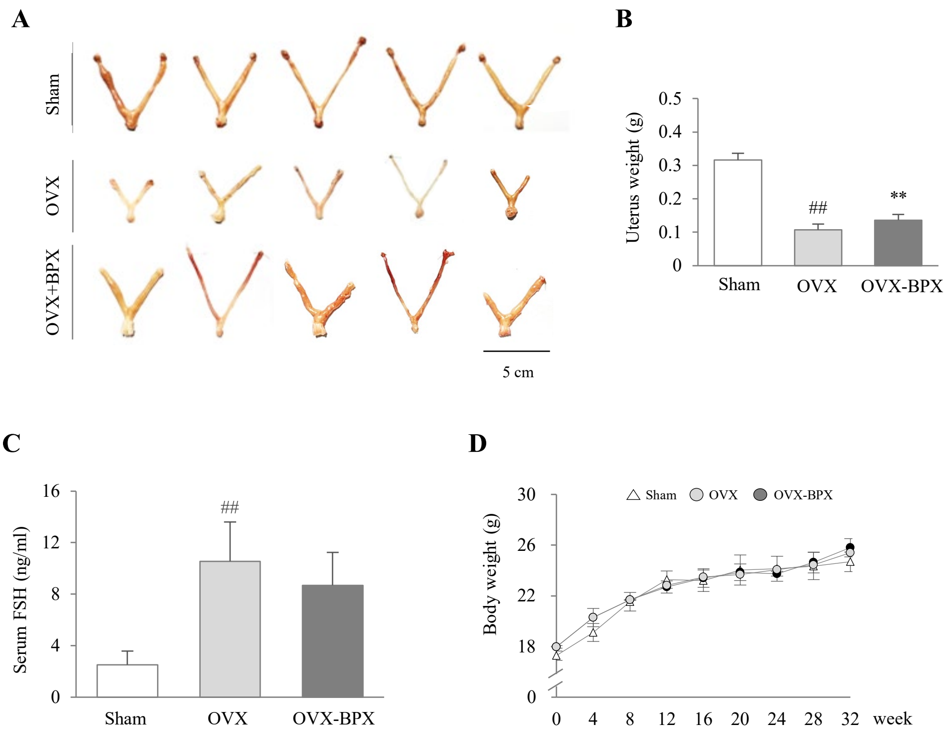

Supplementary Figure 1. Effects of BPX on uterus weight and follicle-stimulating hormone level in serum. Representative uterus morphology was photographed (A) and weighed (B), after sacrifice. The levels of FSH in serum were analyzed by ELISA (C). The body weight was recorded every four weeks throughout the experiment (D). The mice were divided into groups according to the treatment: the Sham, OVX, and OVX+BPX groups. The data are expressed as the mean ± SD. # $p < 0.05$, ## $p < 0.01$ compared with the Sham group; * $p < 0.05$, ** $p < 0.01$ compared with the OVX group.

Supplementary figure. 2

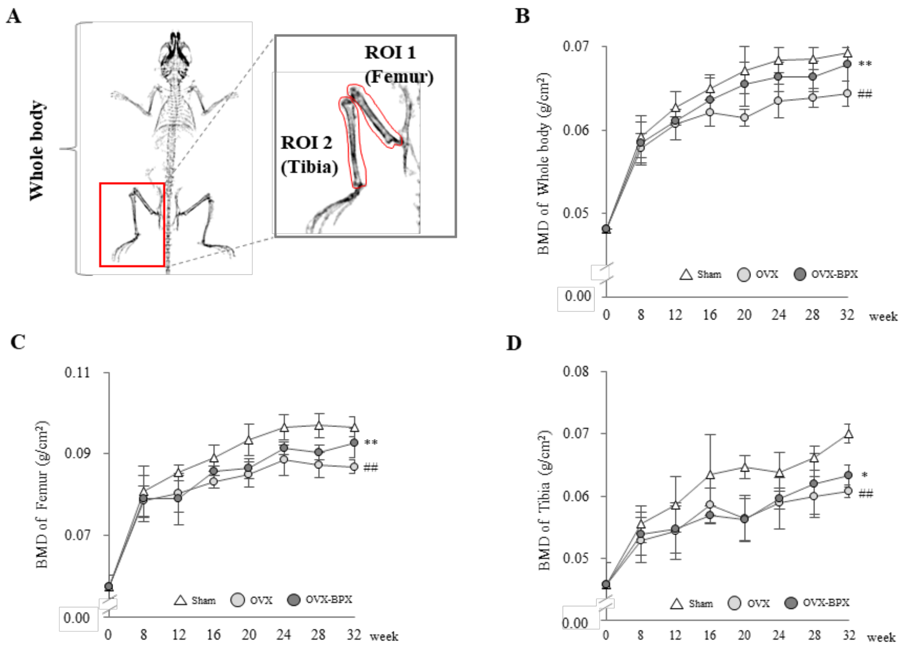

Supplementary Figure 2. Effects of BPX on the alterations in BMD in OVX-induced mice. The BMD scores in the whole body (B), femur (C), and tibia (D), were analyzed for 32 weeks by DXA-drawing region of interest (ROI) boxes surrounding the relevant locations (A) in OVX-induced mice. The mice were divided into groups according to the treatment: the Sham, OVX, and OVX+BPX groups. The data are expressed as the mean ± SD. # $p < 0.05$, ## $p < 0.01$ compared with the Sham group; * $p < 0.05$, ** $p < 0.01$ compared with the OVX group.

Supplementary figure. 3

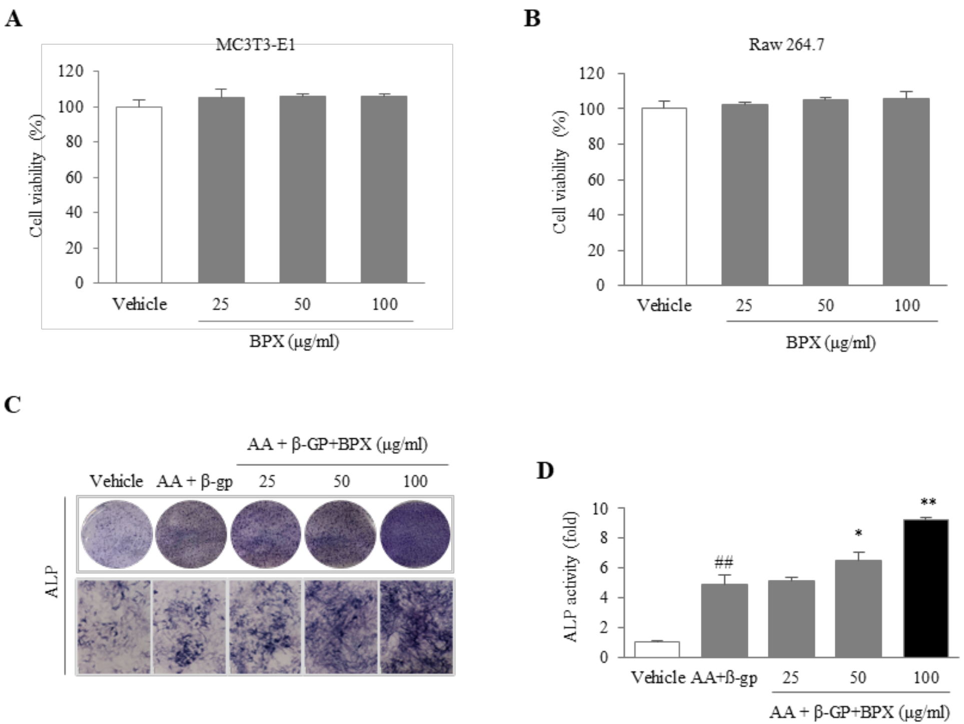

Supplementary Figure 3. Effects of BPX on the bone formation. To confirm the cytotoxicity of BPX, we additionally performed cell viability test using a WST-8 assay kit in MC3T3-E1 (A) and Raw 264.7 cells (B). The MC3T3-E1 cells were treated with β-glycerophosphate (10 mM) plus L-ascorbic acid (50 μg/ml) or BPX (25, 50 and 100 *μ*g/mL) in the condition for osteoblast differentiation, and ALP staining on day 7 (C), and relative ALP activity was quantified on day 7 (D). The data are expressed as the mean ± SD. # $p < 0.05$, ## $p < 0.01$ compared with the Vehicle; * $p < 0.05$, ** $p < 0.01$ compared with the AA + β-gp group.

Supplementary figure. 4

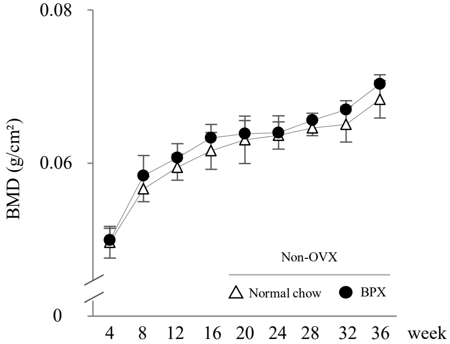

Supplementary Figure 4. Effects of BPX on the alterations in BMD in normal mice. The BMD scores in the whole body for 32 weeks by DXA in normal mice. The mice were divided into groups according to the treatment: the normal chow and BPX groups. The data are expressed as the mean ± SD

Supplementary figure. 5

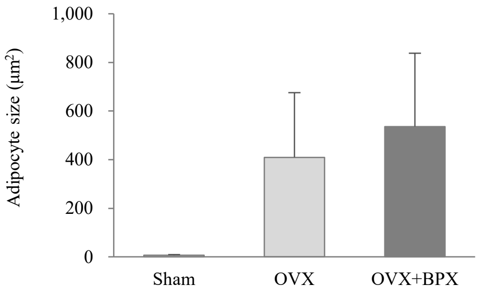

Supplementary Figure 5. Effects of BPX on adipocyte size in OVX-induced mice. The size of adipocytes (μm^2) was measured using image J in OVX-induced mice. The mice were divided into groups according to the treatment: the Sham, OVX, and OVX+BPX groups. The data are expressed as the mean ± SD.
